# Blocking HMGB1/RAGE Signaling by Berberine Alleviates A1 Astrocyte and Attenuates Sepsis-Associated Encephalopathy

**DOI:** 10.3389/fphar.2021.760186

**Published:** 2021-11-15

**Authors:** Jian Shi, Huan Xu, María José Cavagnaro, Xingmei Li, Jia Fang

**Affiliations:** ^1^ Department of Spine Surgery, The Third Xiangya Hospital, Central South University, Changsha, China; ^2^ Department of Hematology and Critical Care Medicine, The Third Xiangya Hospital, Central South University, Changsha, China; ^3^ Department of Laboratory Medicine, The Third Xiangya Hospital, Central South University, Changsha, China; ^4^ College of Medicine-Phoenix, University of Arizona, Phoenix, AZ, United States; ^5^ Department of Forensic Science, School of Basic Medical Sciences, Central South University, Changsha, China; ^6^ Key Laboratory of Sepsis Translational Medicine of Hunan, Central South University, Changsha, China; ^7^ The Department of Neurology, the Second Xiangya Hospital, Central South University, Changsha, China

**Keywords:** berberine, sepsis-associated encephalopathy, HMGB1/RAGE signaling, cognitive impairment, inflammatory cytokines, neuropharmacology, women in neuroscience

## Abstract

As a life-threatening multiple organ dysfunction attributable to maladjusted host immune responses to infection, sepsis is usually the common pathway to serious prognosis and death for numerous infectious diseases all over the world. Sepsis-associated encephalopathy (SAE) is frequently complicated by septic conditions, and is one of the most important reasons for increased mortality and poor outcomes in septic patients which is still an urgent clinical problem need to be solved. In this research, a conspicuously discovery of treatment-related translational use for berberine was elaborated. The results revealed that berberine treatment significantly restored cognitive impairment in sepsis mice. Reduced expression levels of TNF-α, IL-1α, and C1qA were exhibited in the hippocampus of the berberine treatment group, and attenuated effect of declining neo-neuron, activation of microglia and astrocytes in the hippocampus of mice with sepsis were also found. Moreover, berberine inhibits microglia-stressed A1 astrocytes by inhibiting HMGB1 signaling was revealed, then the molecular mechanism of HMGB1/RAGE signaling inhibition leads to the better outcome of SAE was elucidated. To summarize, this research indicated that berberine targets HMGB1/RAGE signaling to inhibit microglia-stressed A1 astrocyte and neo-neuron decline, which consequently alleviates sepsis-induced cognitive impairment. Collectively, berberine may serve as potential therapeutic drug and HMGB1/RAGE signaling would be a novel target for medicine development for treating SAE.

## Introduction

Sepsis is one of the most important leading causes of mortality and morbidity worldwide. According to the third international consensus definitions for sepsis and septic shock (Sepsis-3) in 2016, sepsis should be defined as life-threatening organ dysfunction caused by a dysregulated host response to infection (Singer et al., 2016). Approximately half of sepsis patients suffer from encephalopathy, which induced most of cognition damage and changed mentality in intensive care units (ICU) (Liddelow et al., 2017). Sepsis-associated encephalopathy (SAE) is a diffuse brain disturbance that usually occurs following infection in the body with hardly any central nervous system infection. SAE brought greater risks for long periods of cognitive impairments, which contains alteration in visual-spatial abilities, deficits in visual and functional memory with depressive and/or anxiety disorders (Iacobone et al., 2009; Feng et al., 2019). The quality of life of sepsis patients will be harshly affected by all these faultiness. Furthermore, a lot of limitations and difficulties in clinical practical operation strongly impeded the accurate assessment of cognition and sensory functions in sepsis patients. Consequently, SAE has enhanced harmful effects on patients and brought society a heavy and painful burden.

Despite the growing number of studies have focused on the pathophysiology and related molecular mechanisms, however, the precise etiopathogenesis of sepsis as well as SAE still remain obscure. In sepsis, a great amount of pro-inflammatory cytokines and damage associated molecular patterns (DAMPs) released, including tumor necrosis factor-α (TNF-α), interleukin (IL) family and high mobility group box 1 (HMGB1) which lead to further organ dysfunction and multiple cell death (Andersson and Tracey, 2003; Lu et al., 2013; Denning et al., 2019; Feng et al., 2019; Wu et al., 2021). Exposed to all these inflammation related factors usually cause tissue damage in susceptible areas of the brain like hippocampus. Therefore, effective treatment towards related molecular targets and other key mediators in the pathogenesis of sepsis were imperative in avoiding the happening of SAE (Gofton and Young, 2012; Kuperberg and Wadgaonkar, 2017; Zhong et al., 2020).

Recently, with the rapid development in neuropharmacology, plenty of neurotherapeutic natural products were identified, including ginsenoside Rg1, baicalein, curcumin and gastrodin have proved effective in treating SAE through suppression of different signaling pathways (Li et al., 2017; Fu et al., 2018; Alikiaii et al., 2021). Berberine, a quaternary ammonium salt from the protoberberine group of benzylisoquinoline alkaloids found in such plants as Berberis which can be isolated from a variety of plants, has already shown its excellent effect in anti-oxidant, anti-cancer and anti-diabetic treatment (Prajwala et al., 2020; Yuan et al., 2021). In the field of sepsis research, berberine could partly attenuate sepsis-induced multiorgan dysfunction and neutrophil tissue infiltration, it could also prevent intestinal mucosal barrier damage at the early stages of the disease (Li et al., 2015; Pierpaoli et al., 2020). However, whether berberine treatment attenuates SAE and improves cognitive functions after sepsis still remain to be elucidated.

In this study, we aimed to evaluate the effects and the underlying mechanisms of berberine on cognitive deficits induced by caecal ligation and puncture (CLP) in mice, which may facilitate the medicine development for treating SAE.

## Materials and Methods

*Ager* ^*−/−*^ (or *Rage* ^*−/−*^, KOCMP-11596-Ager-B6N-VA, Syagen) and WT C57BL/6 male mice (Hunan SJA Laboratory Animal Co., PRC) (8–10 weeks, 22–25 g) were adopted herein. The animals were kept under a special sterilized status in Central South University (CSU) with normal parameters (22–25°C and a 12-h light-dark cycle). The entire research was accepted and coincided with the protocols of CSU (IRB 2021-S076).

For the CLP pattern, the caecum was subjected to exposure posterior to a 1.5 cm longitude middle line incision in mice under the anesthesia via 2% isoflurance with O_2_. A polymicrobic septic pattern was completed via the ligation of half of the caecum (Mild-grade type with around 40% expected mortality) and the squeezing out of a little egesta from a complete puncture by 18-gauge needles. The caecum was replaced and the stomach was sealed. NC (37°C; 5 ml per 100 g body weight) was given by subcutaneous injection to awaken the animals from anesthesia. Pseudo operation mice were treated with the identical process apart from the ligating and puncture. Ten days posterior to the CLP treatment, open field test, novel object recognition and Morris water maze test assay were completed.

### Open Field Test

The manoeuvrability of the animals was evaluated through Open Field Test before the cognitive assay (Gould et al., 2009). Overall, the animals were placed carefully in a 50 × 50 cm quadrate case, and a 10-min free movement is permitted. The move was recorded, and the overall distance was documented and studied.

### Novel Object Recognition

Novel object recognition (NOR) assay was completed as above mentioned (Antunes and Biala, 2012). In short, the same two items were placed symmetrically with the same distance from the central point and the walls. The animals were permitted to finish the training as above mentioned. Afterwards, the time costed for every item was documented. 24 h later, during the test phase, one of the items was substituted with a new one with diverse appearance. The time costed for every item was documented. Their move was documented and studied, while the difference denotes the rate of sniffer time on the new item to the two items.

### Morris Water Maze Test

The space learning and retention of the animals were evaluated via Morris water maze (MWM) testing (Vorhees and Williams, 2006). In short, a crystal round terrace was placed 1 cm under the water at one of the quadrants. The animals were permitted to be on the terrace for half a minute to retain the surroundings, and were afterwards placed in the water for training. In every trail, the animals were permitted to discover the terrace in 1 min. If they didn’t succeed, they would be led to the terrace and be on it for half a minute. The training was completed 3 times a day for 4 days, where the release quadrant was altered every trial. Then, the terrace was moved away and the animals were placed in the heterolateral quadrant of the terrace. The move was documented in every assay, and the time to the terrace was studied.

### Immune Staining

After the perfusion of DPBS (pH 7.4) and 4% PFA, the cerebra were treated with fixation via 4% PFA overnight. The cerebra were subsequently subjected to dehydration via graded saccharose liquor, treated with embedment in OCT and incessantly sliced into 30 μm cristated slices at 20°C. Blocked by 5% BSA and 0.1% TritonX-100 for 60 min at ambient temperature, the sections were cultivated via the first antibody at 4°C overnight, such as anti-mouse antibody to IBA1 (1:500), GFAP (1:500), DCX (1:500), and NeuN (1:500, Millipore, America). A cultivation of second antibody (1: 500) for 120 min was subjected completed for the sections. We completed 3 times of cleaning via 0.01 M PBS with 0.1% TritonX-100 between every step. The sections were subjected to image formation via the microscope under the identical illuminous intensity and exposal time.

### qRT-PCR

Overall RNA of purified or cultured cells was separated via Trizol (Life Technologies, Gaithersburg, MD) and turned into cDNA through reversal transcription via Reversal Transcription Kit (Cat #k1622, Thermo Fisher Scientific) as per the supplier’s specification. Designed primer pair (mouse TNF-α: 5′-CCC​TCA​CAC​TCA​GAT​CAT​CTT​CT-3′, 5′-GCT​ACG​ACG​TGG​GCT​ACA​G-3′; IL-1α: 5′- CGA​AGA​CTA​CAG​TTC​TGC​CAT​T-3′ 5′-GAC​GTT​TCA​GAG​GTT​CTC​AGA​G-3′; C1qA: 5′-AAA​GGC​AAT​CCA​GGC​AAT​ATC​A-3′ 5′-TGG​TTC​TGG​TAT​GGA​CTC​TCC-3′; C3: 5′-CCA​GCT​CCC​CAT​TAG​CTC​TG-3′; 5′-GCA​CTT​GCC​TCT​TTA​GGA​AGT​C-3′) and TaqMan Universal PCR master mix (Applied Biosystems, America) were adopted for the amplification of targeted cDNA fragments. Standardization was completed as per the magnification of GAPDH. The expressing levels of the targeted genes were described as the fold variations compared with the control group.

### ELISA Quantification of Cytokines

Purchasable kits (88-7324-88 for TNF-α and 88-5019-88 for IL-1α from Invitrogen; WEA747Mu for C1q and SEA399Mu for HMGB1 from Cloud-Clone) were employed to determine the quantity of cytokines in the media or the plasma as per the supplier’s specification.

### Primary Cell Culture

Primary mouse microglia and astrocytes were separated from P3-4 WT pups. Anti-GLAST MicroBead Kit (Miltenyi Biotec, Germany) was adopted to purify astrocytes. After a challenge of LPS (1 μg/ml, InvivoGen) or recombinant HMGB1 (400 ng/ml, Sino Biological) for 3 h, the medium was harvested and applied to astrocytes for 24 h. The TNF-α quantity in the supernatants were measured via ELISA.

### Statistical Analysis

The statistic assay was completed via GraphPad Prism 6.0. The normal distribution of date was assumed based on normality test. The figures were studied via Student’s t test to contrast between the two groups. Contrasts among ≥2 groups were finished via ANOVA with post hoc test. A *p* value <0.05 was deemed as important on statistics. Assays were separately completed ≥3 times while the entire data were expressed as the mean ± SEM.

## Results

### Berberine Alleviates Cognitive Impairment and Neo-Neuron Decline in Septic Mice

**FIGURE 1:**
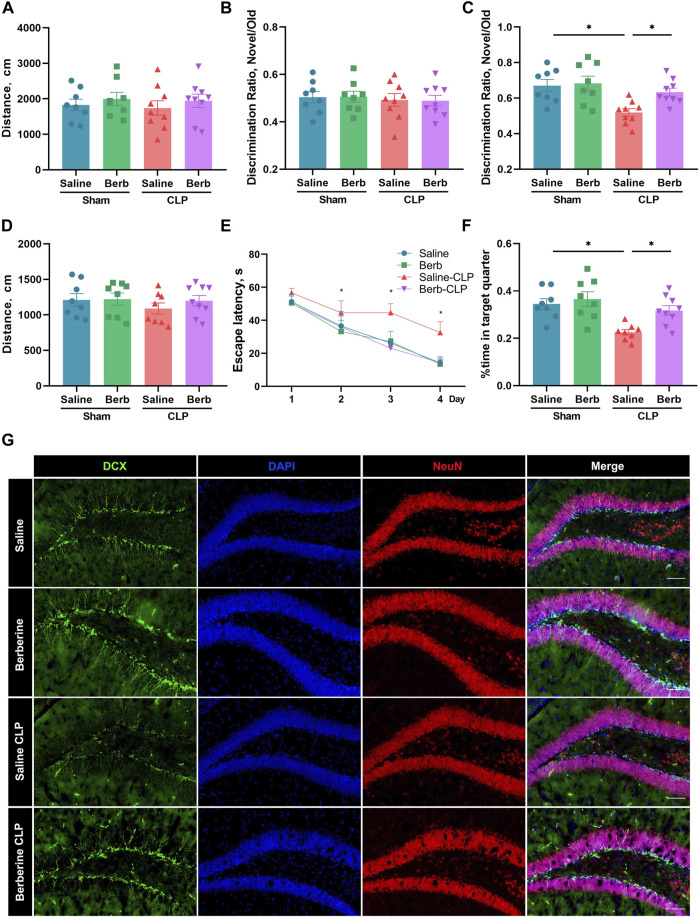
Berberine attenuates cognitive impairment and neo-neuron decline in septic mice. **(A–G)** Mice were administrated with saline or berberine (10 mg/kg) 30 min prior to a challenge of cecal ligation and puncture. Traveling distance in Open Field Test **(A)**, discrimination ratio of training phase **(B)** and test phase **(C)** in Novel Object Recognition (*n* = 8, 8, 9, 9, respectively), and swimming distance **(D)**, escape latency of training phase **(E)** and time spent in the target quarter of test phase in Morris Water Maze (*n* = 8, 8, 8, 9, respectively). **(F)** Immunofluorescent staining of DCX (Green, 200×) and NeuN (Red, 200×) in the hippocampus (Scale bar = 100 μm). * indicates *p* < 0.05 in the comparisons of saline-treated CLP group with other groups.

To determine the effects of berberine on SAE, mice were subjected to OFT to assessing the mobility prior to the tests of NOR and MWM. The results documented that no significant difference was found between groups (Figure 1A), indicating that mobility was restored and would not interrupt the test of cognitive function. In the training phase of NOR, mice in all groups showed similar discrimination ratio (DR) and the DRs were around at 0.5, which means that mice have equal chance to access the two objects and the setting of NOR is appropriate (Figure 1B). When the novel object was set in the testing phase of NOR, as predicted, sham mice administrated with either saline or berberine had a memory of the object with a DR over 0.5, whilst mice with CLP alone lost the memory of the old object and consequently had a DR around 0.5. Administration of berberine significantly restored the decline of cognitive function cause by CLP (Figure 1C). In addition, swimming capacity in MWM was not significantly different between groups (Figure 1D). In compared with negative setting of sham mice, learning ability as assessed in the training phase of MWM was significantly dampened in the mice with CLP alone, but was reversed by administrating berberine (Figure 1E). Similarly, CLP-decreased spatial memory determined in the testing phase of MWM was significantly restored by the treatment of berberine (Figure 1F). Collectively, berberine significantly restored cognitive deficits in mice challenged with sepsis.

It is reported that decline of neo-neurons is associated with cognitive impairment in a series of encephalopathy. To test the effects of berberine on sepsis-induced neo-neuron decline, Doublecortin (DCX) of the hippocampus of mice was visualized using immunofluorescence (Reiner et al., 2006; Klempin et al., 2011). The results showed that sepsis significantly decreased neo-neurons, which was reversed by the administration of berberine (Figure 1G). Thus, berberine may protect against sepsis-induced cognitive impairment by restoring neo-neuron decline.

### Berberine Extinguishes Inflated Microglia and Astrocytes in Septic Mice

**FIGURE 2:**
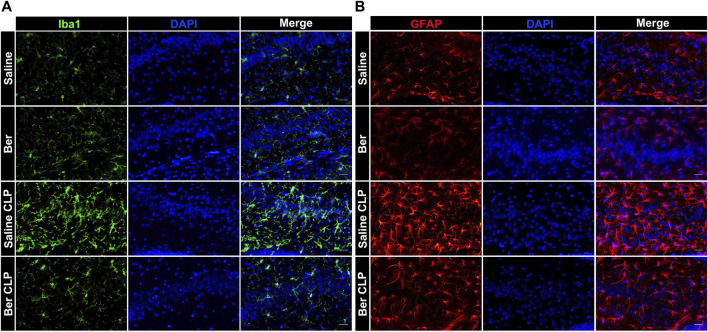
Berberine alleviates activation of microglia and astrocytes in the hippocampus of mice with sepsis. **(A, B)**. Immunofluorescent staining of microglia (Iba1, Green, 400×) and astrocytes (GFAP, Red, 400×) in the hippocampus of mice administrated with saline or berberine (10 mg/kg) 30 min prior to a challenge of cecal ligation and puncture (Scale bar = 100 μm).

It has been shown that the activation of microglia and astrocytes is implicated in sepsis and contributes to impairment of neurogenesis (Liddelow et al., 2017). To determine the underlying mechanism by which berberine protests against SAE, the microglia (Iba1+) and astrocytes (GFAP+) of the hippocampus of mice were determined. Laparotomy alone or with administration of berberine did not have effects on the activation of microglia and astrocytes. By contrast, sepsis robustly activated microglia (Figure 2A) and astrocytes (Figure 2B) in the hippocampus, which was reversed by the treatment of berberine. Thus, the protection of berberine may attribute to the effects on suppressing activation of microglia and astrocytes in septic mice.

### Berberine Alleviates Microglia-Stressed A1 Astrocytes by Inhibiting HMGB1 Signaling

**FIGURE 3:**
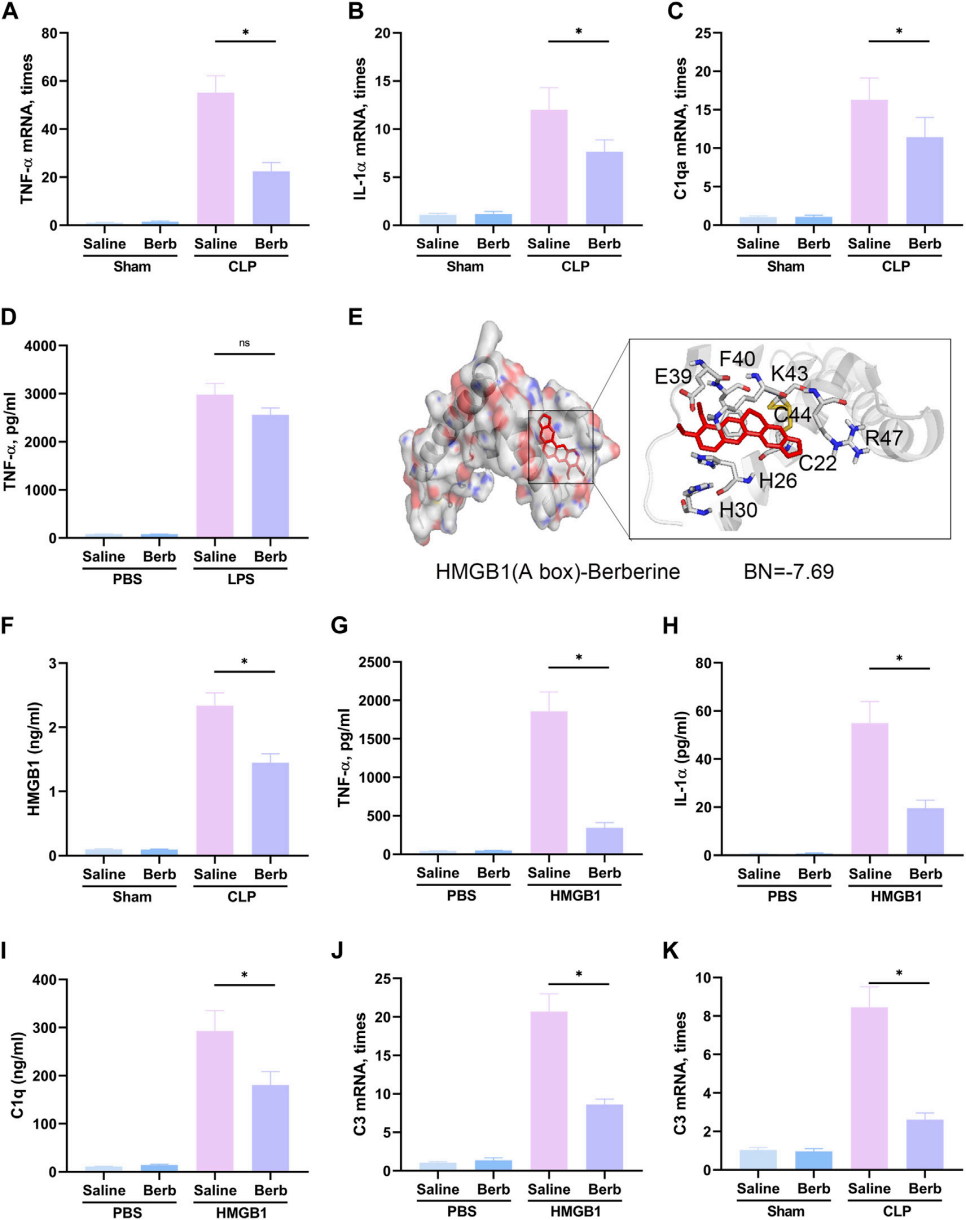
Berberine inhibits microglia-stressed A1 astrocytes by inhibiting HMGB1 signaling. **(A–C)**. Expression of TNF-α **(A)**, IL-1α **(B)**, and C1qA **(C)** in the hippocampus of mice administrated with saline or berberine (10 mg/kg) 30 min prior to a challenge of cecal ligation and puncture. **(D).** Medium level of TNF-α in the microglia treated with beriberine (5 μM) or saline prior to a challenge of LPS (1 μg/ml) or not. **(E)**. Binding of berberine and HMGB1 in the analysis of molecular docking. **(F)**. Plasma levels of HMGB1 in mice administrated with saline or berberine (10 mg/kg) 30 min prior to a challenge of cecal ligation and puncture. **(G–I)**. Medium level of TNF-α **(G)**, IL-1α **(H)**, and C1q **(I)** in the microglia treated with beriberine (5 μM) or saline prior to a challenge of recombinant HMGB1 (400 ng/ml) or not. **(J)**. Expression of C3 (an A1 astrocyte-specific gene) in astrocytes co-cultured with microglia that were treated with beriberine (5 μM) or saline prior to a challenge of recombinant HMGB1 (400 ng/ml) or not. **(K).** Expression of C3 in the hippocampus of mice administrated with saline or berberine (10 mg/kg) 30 min prior to a challenge of cecal ligation and puncture. * indicates *p* < 0.05 in the comparisons of saline-treated with berberine-treated groups.

The astrocytes could be classified into A1 and A2 phenotypes, in which A1 astrocytes exudes toxic factors that could kill mature neurons and oligodendrocytes, have been proved to be involved in a lot of different neurological diseases (Li et al., 2020; Ren et al., 2020) which is a very important factor in SAE. To further assess the mechanisms of berberine-mediated protection, expression levels of TNF-α, IL-1α, and C1qA, essential microglia-secreted cytokines in the formation of A1 astrocytes, were determined in the hippocampus of mice. The results demonstrated that the expression of TNF-α, IL-1α, and C1qA was remarkably increased in the mice challenged with CLP (Figures 3A–C). Administration of berberine significantly restored the highly expressed cytokines. LPS was reported to be the major stimulator of microglia-stressed A1 astrocytes. Here, LPS significantly boosted the level of TNF-α in primary microglia, whereas berberine slightly but not significantly decreased the augment of TNF-α (Figure 3D), indicating berberine may not inhibit TLR4-mediated TNF-α expression. Given that HMGB1/RAGE signaling is a pivotal upstream of TNF-α and implicated in SAE, the binding of berberine and HMGB1 was assessed using molecular docking. Berberine had a high binding energy with HMGB1 (−7.69, Figure 3E). In addition, berberine significantly restored the augment of plasma HMGB1 in CLP-challenged mice (Figure 3F). In in-vitro model, treatment of berberine significantly attenuated HMGB1-upgraded TNF-α, IL-1α, and C1q levels in primary microglia (Figures 3G–I) and HMGB1-mediated A1 astrocytes (C3, A1 astrocyte-specific, Figure 3J) in microglia and astrocyte co-cultured system. Moreover, in *in-vivo* model, berberine had inhibitory effects on C3 expression (Figure 3K). Taken together, it can be concluded that berberine reduces pro-inflammatory cytokine release and A1 astrocytes activation by inhibiting HMGB1 signaling.

### Berberine Restored Sepsis-Induced Cognitive Impairment and Neo-Neuron Decline by Inhibiting HMGB1/RAGE Signaling

**FIGURE 4:**
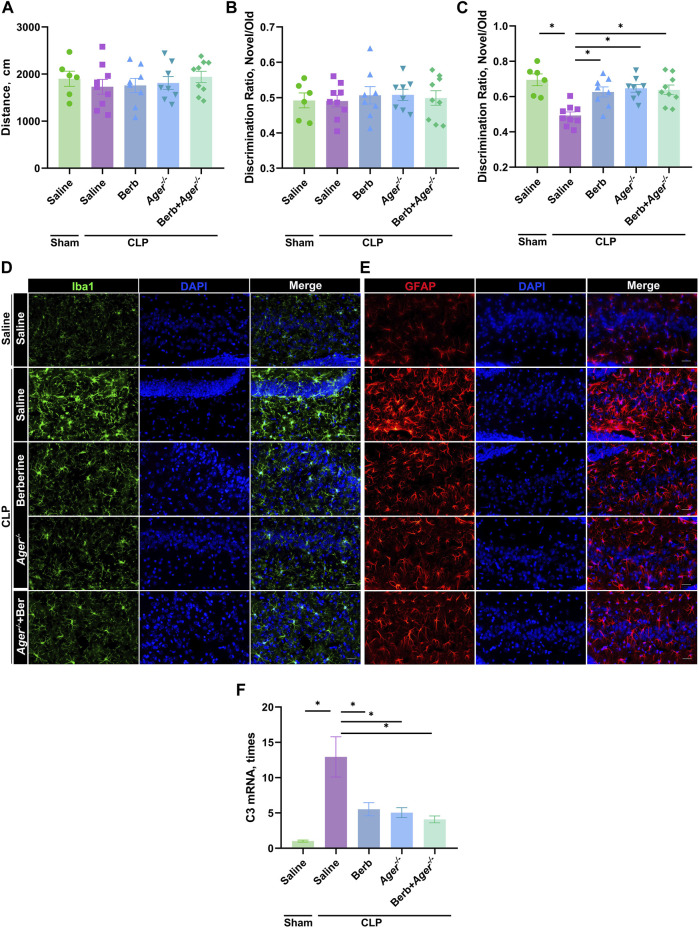
Berberine improve sepsis-associated encephalopathy by inhibiting HMGB1/RAGE signaling. **(A–F).** Ager^−/−^ or WT mice were administrated with saline or berberine (10 mg/kg) 30 min prior to a challenge of cecal ligation and puncture. Traveling distance in Open Field Test **(A)**, discrimination ratio of training phase **(B)** and test phase **(C)** in Novel Object Recognition (*n* = 6, 9, 8, 8, 9, respectively). **(D)** and **(E)**. Immunofluorescent staining of microglia (Iba1, Green, 400×) and astrocytes (GFAP, Red, 400×) in the hippocampus of mice (Scale bar = 100 μm). **(F)** C3 expression levels in the hippocampus of mice * indicates *p* < 0.05 in the comparisons of saline-treated CLP group with other groups.

RAGE is the receptor of HMGB1 and mediates HMGB1 downstream signaling (Rauvala and Rouhiainen, 2007; van Zoelen and van der Poll, 2008; Deng et al., 2018). To further confirm the effects of berberine on HMGB1/RAGE signaling in the protection of SAE, WT or *Rage* ^*−/−*^ mice administrated with berberine or not were subjected to CLP or laparotomy alone, followed by cognitive tests. Similar moving distance between groups in OFT confirmed the sufficient mobility restoration for cognition tests (Figure 4A). Similar DRs around 0.5 between groups in the training phase indicated appropriate experiment setting of NOR (Figure 4B). In the testing phase of NOR, similar to administration of berberine, deficiency of *Rage* significantly restored the memory loss of old objects in septic mice (Figure 4C). Administration of berberine in Rage^−/−^ mice did not further improve the cognitive restoration (Figure 4C). Moreover, activation of microglia and A1 astrocytes had similar trend (Figures 4D–F). Thus, berberine protects against SAE by inhibiting HMGB1/RAGE signaling.

## Discussion

In terms of morbidity and mortality among various serious diseases, sepsis imposes a substantial global burden and needs to be diagnosed and treated as soon as possible. The pathogenesis of SAE is inextricably linked to neuroinflammation, with uncontrolled inflammation as one of its main characteristics. Neuroinflammation is the major culprit for dysfunction and massive apoptosis of neurons, endothelial cells, and microglial cells (Iacobone et al., 2009; Liddelow et al., 2017). Both local and peripheral inflammation are brought out by the activation of resident brain immune cells, particularly the astrocytes. Previous research have proved that the induction of neuroinflammatory response and worse clinical prognosis are as a consequence of septic complications. And just as importantly, activated microglia can release a number of inflammatory mediators, the most important of which in SAE are TNF-α, IL-1α, and C1qA. These inflammatory mediators will further facilitate the formation of neuron-toxic A1 astrocytes and aggravate neuroinflammation (Xu et al., 2019; Lu et al., 2020). Then sustained damage of endothelial cells caused neuroinflammation leads to cerebral perfusion disorder, which makes SAE a sophisticated and intractable problem. Without any doubt, early diagnosis and treatment of SAE are extremely important.

Previous investigations have demonstrated that berberine and its correlative derivate exhibited extensive anti-inflammation and anti-tumor roles, which could be a new potential therapeutic drug (Kumar et al., 2015; Prajwala et al., 2020). Herein, our study revealed that the utilization of berberine before sepsis could avoid the progress of cognition function disorder in CLP-caused septic mice (Lu et al., 2020). More studies unveiled that such protection effect for cognition damage was closely associated with the decrease of pro-inflammation cell factors and the mitigation A1 astrocyte (Sun et al., 2019; Liu et al., 2020). More detailed mechanisms of those anti-inflammation roles of berberine have not yet been fully elucidated. Numerous signal pathways, such as CCR2 expressing in neutrophilic cells, TLRs, NF-ΚB, and PPARγ, are targets of berberine *in vitro* or vivo (Xu et al., 2017; Wang et al., 2018). The increasing beneficial berberine effect towards SAE and the precise mechanisms require further investigations.

Current studies have demonstrated that HMGB1, the late mediating factor in sepsis pathogenesis and an essential factor that mediates cognitive impairment in sepsis survivors (Chavan et al., 2012), triggers and sustains the inflammatory response by inducing cytokine release and recruiting leucocytes (Magna and Pisetsky, 2014). These characteristics make extracellular HMGB1 a key molecular target in multiple illnesses. Many approaches have been adopted to suppress HMGB1. Significantly, HMGB1 can activate not only the receptor of advanced glycation endproducts (RAGE) but also other receptors, particularly the Toll-like receptors 2/4 (Lotze and Tracey, 2005). It was also demonstrated that the process of HMGB1 endocytosis requires the RAGE and dynamin-dependent signaling, which in turn influences macrophage pyroptosis during endotoxemia (Xu et al., 2014). And further study has established that cell surface-expressed RAGE could bind to extracellular HMGB1-LPS complexes which will be endocytosed to the endolysosomal compartment (Deng et al., 2018). Moreover, another study has demonstrated that anti-HMGB1 therapeutic modalities caused an extra survival benefit in high-dose endotoxin injected RAGE-deficient mice, which indicating that HMGB1 acts partially through RAGE during septic shock process (Abeyama et al., 2005). These research evidenced that RAGE-mediated internalization is an essential process in Gram-negative sepsis. Additionally, although the HMGB1/RAGE signaling still remains a potential yet promising therapeutic target in SAE, more studies will be required before we know what the functions of RAGE in critical organ disorder implicated in the etiopathogenesis of sepsis and SAE.

Our study results indicated that preprocessing with berberine uplifted the cognition damage by suppressing the quantity of inflammatory events of cell factors, astrocyte activation and neo-neuron decrease in the cerebrum of SAE mice, which revealed that berberine could serve as an underlying medicine for SAE later on. While in clinical and translational studies, berberine is an over-the-counter medicine with verified efficacy to cure enterorrhea and enterogastric dysfunction. Numerous berberine derivates are prepared and assessed for the treatment of multiple illnesses. If berberine and derivate therapy before septic diseases exhibit effectiveness in preventing cognition damage remains elusive and requires more investigations. No matter berberine failed in clinic studies on septic cases or not, it’s one of the valid medicines in preventing cognition damage in pathophysiological research in mice and certain studies on the internal diversities between murine sepsis and mankind septic diseases which require investigations in the near future. The main limitations of the study are the lack of physical confirmation of theoretical binding simulation of berberine with HMGB1 and the assumption of this decreasing HMGB1 binding to RAGE. The long-term safety and efficacy of berberine on SAE need to be further validated in controlled clinical trials which is also another limitation and application insufficiency of this study.

## Conclusion

Overall, our research provides new promising drugs berberine in avoiding the establishment of SAE. Berberine significantly repressed the activation of microglia as well as astrocytes and the decline of natal-neurons and consequently improved cognitive functions in septic mice by blocking HMGB1/RAGE signaling. Hence, berberine marks an underlying HMGB1-targeting medicine for SAE mice, whereas the effectiveness in mankind cases clinically requires more investigations nonetheless.

## Data Availability

The original contributions presented in the study are included in the article/Supplementary Material, further inquiries can be directed to the corresponding author.
